# A Systematic Literature Review of Complementary and Alternative Veterinary Medicine: Laser Therapy

**DOI:** 10.3390/ani13040667

**Published:** 2023-02-14

**Authors:** Darryl L. Millis, Anna Bergh

**Affiliations:** 1 Department of Small Animal Clinical Sciences, College of Veterinary Medicine, University of Tennessee, Knoxville, TN 37996, USA; 2 Department of Clinical Sciences, Swedish University of Agricultural Sciences, SE 750 07 Uppsala, Sweden

**Keywords:** light therapy, laser therapy, therapeutic laser, cold laser, low level laser, photobiomodulation

## Abstract

**Simple Summary:**

Light therapy, or photobiomodulation, is a collective name for methods where tissue is irradiated with different types of light, with the aim of stimulating healing. The group includes methods such as laser, Light Emitting Diode (LED) light, ultra and infrared light, and tanning beds. In animals, the main indications for light treatment are musculoskeletal injuries, neurological diseases, wounds, and pain. Despite being frequently used, there is no consensus regarding the optimal treatment protocols for light therapy nor its clinical efficacy. Therefore, the aim of this systematic review was to evaluate the documented clinical effects of light therapy, with a focus on laser and LED light, in horses, dogs, and cats. The undertaken systematic literature review found gaps in the scientific documentation. Conflicting study results and unclear application for clinical use are explained by the wide variety of treatment parameters used in the analyzed studies, such as wavelength, laser class, dose, and effect, as well as the frequency and duration of treatment. Some beneficial effects have been reported during treatment with light therapy; however, the published studies also have limited scientific quality regarding these therapies, with a high or moderate risk of bias.

**Abstract:**

Light therapy, or photobiomodulation, is a collective name for methods where tissue is irradiated with different types of light, with the aim of stimulating healing. Despite being frequently used, there is no consensus regarding the optimal treatment protocols for light therapy, nor its clinical efficacy. A systematic literature review was conducted, searching the relevant literature regarding light therapy in three databases, published between 1980–2020. The risk of bias in each article was evaluated. Forty-five articles met the inclusion criteria; 24 articles were regarding dogs, 1 was regarding cats, and the rest were regarding horses. The indications for treatment were musculoskeletal and neurologic conditions, skin disease and wounds, and pain. The literature review showed conflicting study results and unclear application for clinical use. This can be explained by the wide variety of treatment parameters used in the searched studies, such as wavelength, laser class, dose, and effect, as well as the frequency and duration of treatment. Although some beneficial effects were reported for light therapy, the studies also had limited scientific quality regarding these therapies, with a high or moderate risk of bias.

## 1. Introduction

Light therapy, or photobiomodulation, is a collective name for methods where tissue is irradiated with different types of light, with the aim of stimulating healing. The group includes methods such as laser, Light Emitting Diode (LED) light, ultra and infrared light, and tanning beds. In humans, light therapy has been used for decades to treat various conditions such as depression, skin disease and wounds, and pain, as well as to promote the healing of musculoskeletal tissues [1]. In animals, the main indications are musculoskeletal injuries, neurological diseases, wounds, and pain [2].

Different light sources have different properties. For example, the light emitting from a laser is monochromatic, is collimated, and is of one wavelength. This is in contrast to ordinary light, with many different noncollimated wavelengths. One difference between laser light and LED light is that laser light waves are coherent, with the wavelengths in phase in space and time.

The mechanism behind light therapy is not clearly understood. Light particles, called photons, are transmitted to tissues. One hypothesis for light’s mechanism of action is that photons are absorbed by cell mitochondrial chromophores, which leads to a photodissociation of inhibitory nitric oxide from cytochrome C oxidase. This is followed by the enhancement of electron transport, enzyme activity, and ATP production, parameters concerned with cell proliferation and tissue repair [3]. 

In practice, many different types of light-emitting sources and protocols are used, with different settings of wavelength, irradiated area, intensity, and treatment time. Most commonly, does are below 50 J/cm^2^ [3]. Increasingly, higher power class IV lasers are used in small animal practice, with up to 15 watts/cm^2^ continuous laser and pulsed lasers with up to 270 watts/cm^2^ peak power being used. Further adding confusion to any discussion of therapeutic lasers are terms such as the type of laser (CO2, HeNe, GaAs, and GaAlAs), the use of either single or multiple wavelengths, and cluster probes which use several different photon emitting devices in a single unit. Despite being frequently used in animals, there is no consensus regarding optimal treatment protocols nor its clinical efficacy for the treatment of various conditions. Therefore, the aim of this systematic review was to evaluate the documented clinical effects of light therapy, with a focus on laser and LED light, in horses, dogs, and cats. 

## 2. Materials and Methods

The overall outline of this systematic review adhered to the Cochrane guidelines regarding how to perform a systematic review [4], as adapted by the Swedish Agency for Health Technology Assessment and Assessment of Social Services (SBU) in its methodological handbook [5]. 

### 2.1. Review Topic/Research Question

To assess the evidence for the clinical efficacy of laser and LED therapy used in horses, dogs, and cats.

### 2.2. Search Strategy

Professional librarians searched the literature in the databases Web of Science Core Collection, CABI, and PubMed (1980–2020) in August 2020. The keywords were terms relevant to dog OR cat OR horse, AND veterinary medicine OR veterinarian, AND therapy * OR treatment *. These keywords were combined with terms related to light therapies, i.e., laser therapy, therapeutic laser, cold laser, low-level laser or photobiomodulation, and light emitting diode. 

### 2.3. General Inclusion and Exclusion Criteria

The included studies were to be original research, which was published in a peer-reviewed journal between years 1980–2020. Primarily observational studies that would have only one method studied per treatment group were included. Experimental studies could also be included when the study mimicked a clinical situation. The subject species were to be either canine, feline, or equine. 

Textbook chapters, conference proceedings, abstracts, opinion notes, review articles, and case reports (subject number < 5) were excluded. Mechanism of action experimental studies were also excluded, along with studies that used multiple treatments simultaneously per intervention group.

### 2.4. Study Selection and Categorisation

All screening was performed based on journal title, publication title, or abstract. Citations identified were imported into Endnote (X9.3.3, 2018) and duplicates were removed. A single author (DM) applied inclusion and exclusion criteria to all publications. 

In the screening phase, articles of possible relevance for the review, and articles describing one type of intervention in cats, dogs, or horses were selected for full text reading. A therapeutic intervention was defined as an intervention intended to reduce the signs, severity, or duration of a clinical condition. After the first stage of screening, articles deemed potentially relevant were accessed. For each study, the following key descriptive items were tabulated using templates modified after SBU [5]: first author, year of publication, study design, study population, intervention, and type of control, outcome, and relevance (external validity). 

Assessment of the risk of bias (scientific quality) of each article was performed in accordance with the Cochrane [4] and SBU [5] guidelines. The assessment was based on the following items: study design, statistical power, deviation from planned therapy, loss to follow-up, type of outcome assessment, and relevance. In the assessment of observational studies, risk of confounding was also included. The writing of the paper has been conducted following the PRISMA 2022 checklist and the study has not been registered in PROSPERO since it is not for human health.

## 3. Results

**Figure 1:**
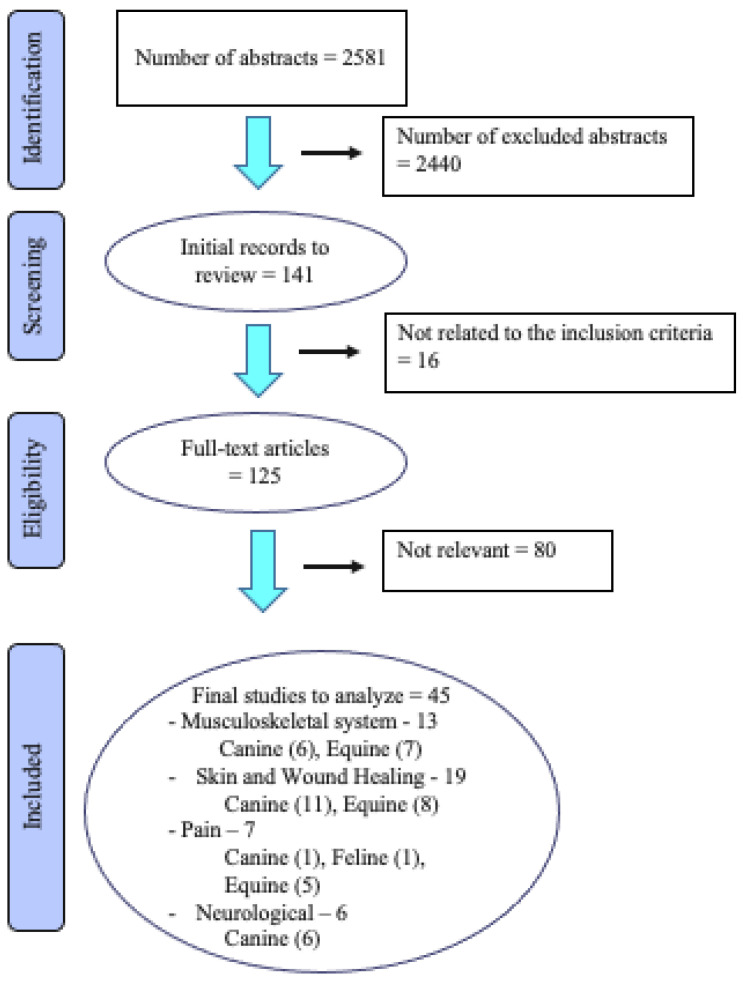
Flow diagram of the stages of the selection process used for identification of studies eligible for final analysis.

A total of 2581 abstracts were identified from the three electronic databases (Figure 1). Duplicates were removed and the abstracts were re-evaluated using the inclusion criteria. A total of 125 publications were studied in detail. Regarding laser treatment of the musculoskeletal system, 50 studies were identified, of which 28 were dog studies, 22 were on horses, and none were cat studies. Thirteen of these met the inclusion criteria. Regarding the effects of laser on skin and wound healing, 44 abstracts were identified, of which 32 were dog studies, 12 were studies of horses, and none were regarding cats. Nineteen of these met the inclusion criteria. In the treatment of pain with laser, three studies were regarding dogs, one was regarding cats, and six studies were on horses. Seven of these were included. Of 20 dog studies regarding laser treatment for various neurological conditions, 6 studies met the inclusion criteria. A horse study was identified; however, it did not meet the inclusion criteria. The studies that did not meet the inclusion criteria were case reports, review articles, method articles, or had phototherapy as part of a combination of treatments, which made the evaluation of phototherapy as a single treatment impossible.

### 3.1. Study Quality

In general, the quality of the studies was low. In many cases, details regarding laser application were missing or inadequate, controls groups were not included, or other interventions were administered along with laser therapy.

### 3.2. General Clinical Indications

Photobiomodulation is used in a variety of applications in veterinary medicine, including musculoskeletal conditions, skin and wound healing, neurological conditions, pain, and a variety of other indications.

### 3.3. Musculoskeletal Conditions

#### Intervention, Control and Clinical Effects

Of the 13 articles on musculoskeletal conditions, 8 had a low risk of bias, 3 a moderate, and 2 a high (Table 1). Of the six studies which evaluated laser for musculoskeletal conditions in dogs, four were randomized, controlled trials (RCT), and three of these showed possible benefits. A study of laser for elbow arthritis indicated improvement in subjective parameters and reduction in the use of non-steroidal anti-inflammatory drugs, evaluated by the dog owner [6]. The laser dose was based on body weight. Three studies evaluated the use of laser treatment in dogs that underwent a tibial plateau leveling osteotomy (TPLO) for cranial cruciate ligament injury. A preoperative treatment was mildly effective in improving post-operative weight bearing, evaluated by force platform measurement [7]. A larger RCT study indicated improvement in lameness via a subjective scoring system; however, no objective methods were used [8]. A study of dogs treated for eight weeks after TPLO used a combination of subjective and objective measures. The study found no significant differences between the treated and placebo groups, with better results observed for the placebo group than the treated dogs at several time points [9].

A research study on osteogenesis reported positive effects of laser treatment on bone formation and healing time of the osteotomy [10]. Interestingly, the TPLO studies also evaluated bone healing, and none of them showed a positive effect. An uncontrolled study of laser treatment for osteomyelitis, after unsuccessful treatment with antibiotics, indicated a positive result in most dogs; however, long-term follow-up was not performed [11].

Seven of the horse studies were RCT. Two studies evaluated carbon dioxide laser for acute traumatic arthritis of the fetlock joint. One study, without an untreated control group, compared betamethasone and hyaluronan with carbon dioxide laser for acute traumatic arthritis, with a more favorable recovery in the laser-treated group [12]. A smaller study, which evaluated laser treatment compared to a control group, showed no differences in subjective lameness assessment, objective movement analysis with accelerometry, and analysis of inflammatory markers in the synovial fluid [13].

Other equine studies largely evaluated the effect of laser on tendon or ligament injuries. A clinical study of horses with ligament or tendon injuries indicated improved edema, lameness, and lesion size; however, no differences in healing were detected with objective ultrasound evaluations [14]. A retrospective study of a large number of horses with superficial digital flexor tendon injuries showed no advantage of laser treatment compared to conservative treatment [15].

Other studies evaluated the penetration ability or thermal effects of lasers on equine tissue. An experimental study of carbon dioxide laser showed increased skin perfusion and temperature in treated horses, with the biggest changes occurring in horses with clipped hair [16]. However, there were no temperature or perfusion differences in deeper tissues. Another study of laser application also showed increased skin temperature, which was assessed with a thermal imager [17]. A study of near-infrared lasers on cadaveric superficial digital flexor tendons indicated poor light penetration into the tissue [18].

### 3.4. Skin and Wound Healing

#### Intervention, Control and Clinical Effects

Of the 19 articles on skin and wounds, 11 had a low risk, 6 had a moderate, and 2 had a high risk of bias (Table 2). Of the 10 dog studies, one randomized controlled trial (RCT) showed potential benefit in wound healing properties. Most of the studies evaluated wound healing or absorbance of laser energy in the tissue. Three well-controlled blinded studies showed no effect of laser treatment on wound healing in surgically-created sutured or open wounds [19,20,21], while one study showed a slightly faster healing time with improved scarring after laser treatment [22]. One study on infected wounds showed no effect of laser treatment [23], while another that used blue light showed improvement of pyoderma in dogs [24]. A study of pedal pruritus due to atopy showed no effect of laser treatment [25].

Studies of laser penetration indicated that if the laser head has contact with the skin, there is better light penetration than when applied without contact [26]. Other aspects that improved light penetration in tissues were clipped hair, and lighter skin color [27]. Most laser energy was found to be absorbed by the superficial tissues. Other studies included the use of lasers to treat aural hematomas [28] and alopecia [29]; these studies indicated a positive effect; however, the design of the studies used subjective outcome measures or had inadequate controls, which left interpretation of the results questionable.

All eight of the horse studies were RCT, with most studies being either tissue penetration of laser light or wound healing, and with two wound healing studies showing no effect and two showing some positive effects. Two well-controlled studies showed no effect of laser on wound healing [30,31], while two indicated an improvement in wound healing of tissue in the pharynx [32] and granulation tissue on the extremities compared with bandages or basic liniments [33]. One study suggested that a very high doses of laser energy may damage tissues [34] (Bergh A, 2007). Three studies evaluated laser penetration into tissues, with most laser energy absorbed by very superficial tissues, similar to the canine studies [35,36,37]. Clipping and cleansing the skin improved penetration, and one study showed better penetration in light horses [37], while another showed no effect of skin color [36].

### 3.5. Pain

#### Intervention, Control and Clinical Effects

In the treatment of pain with laser, three dog, one cat, and six horse studies were identified (Table 3). Of these, one dog, one cat, and five horse studies met the inclusion criteria, and of these seven articles, none had a low risk, three had a moderate, and four had a high risk of bias. A study of dogs undergoing treatment for pain after ovariohysterectomy with laser acupuncture suggested an improved pain relief compared to meloxicam [38]. A similar study of pain relief after ovariohysterectomy was also performed in cats [39]. Although laser acupuncture and electroacupuncture were similar in the treatment of pain, both treatments required fewer rescue analgesics compared to placebo. Three horse studies evaluated laser treatment for back pain. one study had no controls; therefore, no conclusions could be drawn about its effect [40]. The other two suggested positive results regarding the use of laser for the treatment of back pain [41,42]. An uncontrolled study of laser treatment for laminitis indicated an improvement; however, no conclusions could be drawn [43]. Another laser study in horses treated with epidural anesthesia suggested that laser treatment prolonged analgesia with concomitant epidural anesthesia [44].

### 3.6. Neurological Conditions

#### Intervention, Control and Clinical Effects

Of 20 canine studies regarding photobiomodulation for neurologic conditions, six met the inclusion criteria, with one having a low, three a moderate, and two a high risk of bias (Table 4). Only one of the four studies of laser for the postoperative treatment of intervertebral disk disease [45,46,47] showed a positive effect [48]. However, the dose of laser used in that study was unclear. Another experimental study of sciatic nerve injury indicated an improvement in EMG activity after laser treatment [49]. A study of laser use in treating degenerative myelopathy did not have a control group (comparison to a historical control group was used), had other confounding factors that may have influenced the results, and the laser dose used to compare Class III and Class IV lasers was not the same [50].

## 4. Discussion

In this systematic literature review, 45 studies were identified and evaluated in which light therapy was used to treat musculoskeletal and neurologic conditions, skin and wounds, and pain, in dogs and horses, as well as ovariohysterectomy in cats.

Of the 13 articles on musculoskeletal conditions, 4 experimental studies investigated tissue temperature changes and light penetration depths [10,16,17,18]. The studies with a low risk of bias had conflicting results, varying from positive effects on weight distribution and pain scores, to no significant differences. Of the 19 articles on skin and wounds, the majority were experimental studies investigating the healing of surgically-created wounds, skin and hair characteristics affecting laser penetration, and light penetration depths. The studies with a low risk of bias showed no significant differences in quality or rate of wound healing. Of the seven articles on pain, all were clinical studies, with some suggesting some benefit regarding pain relief.

Finally, of the six articles regarding neurologic conditions, laser treatment had mixed benefits. The single study with a low risk of bias reported no significant differences in time to reach recovery or duration of postoperative IV opioid administration following intervertebral disk herniation surgery [46]. One experimental study evaluated the effect of laser therapy after experimental crush sciatic nerve injury and suggested some benefit [49].

The reasons for the studies’ risks of bias, which make it difficult to draw certain conclusions from the literature review results, are several. In the evaluated studies, the small groups of animals were consistently a major source of a high risk of bias, as well as studies where control groups were missing or where people who evaluated/made measurements on the animals were aware of the treatment given. Study groups with individuals of different ages, genders, and breeds could also contribute to a large spread of the results. The design of some of the studies was of insufficient quality to convince the research and veterinary scientific communities to accept (or discard) the therapy. Additionally, a major reason for the difficulty in drawing any firm conclusions is the heterogeneity of the treatment protocols. Despite the majority of studies looking at therapeutic laser therapy, the included studies used different types of lasers, with different wavelengths. Further, there was no agreement regarding dose and treatment time, and in many cases, the treatment parameters were not fully described. Considering the heterogeneity between studies and the low number of studies for each combination of species and indication, pooled statistical analysis with meta-analysis was not feasible. With many small studies, the possibility of publication bias increases; small studies with a negative outcome are less likely to be published [51]. The methods to detect and adjust for publication bias require that there are at least some larger studies to be used as a reference [52]. This made it impossible to conduct any further statistical analyses on the material.

When a method is introduced to veterinary medicine, it is important to know its clinical effects for its proposed indications. Before a sufficient number of studies have been conducted, and a meta-analysis of the effects performed, it is important to have knowledge of the method’s mode of action and application. The results of this literature review suggest that several factors may affect this proposed mechanism of action; the penetration depth of light depends on the wavelength of the laser, if the hair coat is clipped or not, and the type of tissue that is irradiated. The fact that some lasers may cause an increase in tissue temperature, and subsequent increase in blood flow [16,17], may be positive for healing processes; however, it may also pose an increased risk of injury [34]. It is also possible that the influence of light therapy, with a focus on the laser, on tissue effects depends on the total dosage of laser radiation used, and the time and method of radiation. It has been suggested that radiation in the early stages of healing, and repeated radiation over a certain period, could have different amounts of effectiveness on tissues such as bone [53].

## 5. Conclusions

The systematic literature review undertaken in this study found a general lack of quality evidence in the scientific documentation regarding the clinical effects of laser and LED light therapy in horses, dogs, and cats. For most of the indications, there was insufficient scientific evidence for favorable clinical effects; the studies were (a) negative or (b) of insufficient quality, or (c) the results were contradictory or (d) confirmatory results were lacking. Although some beneficial effects have been reported for laser therapy, the conflicting study results and unclear application for clinical use are explained by the wide variety of treatment parameters used in the studies, such as wavelength, laser class, dose and effect, and the frequency and duration of treatment. In many cases, the description of phototherapy use was incomplete, such as the dose used.

Although potential mechanisms of action have been evaluated in tissue and cell culture, as well as some live animal experiments, clinical trials to evaluate the efficacy and proper use of light therapy must be conducted prior to its widespread application for clinical use. Moving forward, multi-institutional studies evaluating a large number of patients with strict inclusion criteria for various conditions and complete description of the application of light therapy are recommended. Additionally, dose titration studies are needed to determine the optimal dose, power, and frequency of light therapy for clinical use.

## Figures and Tables

**Table 1:** Summary of laser and LED therapy based on a systematic review of the published literature: musculoskeletal conditions.

Musculoskeletal Conditions							
Study	Study Design	Control Group	Study Sample	Intervention and Dosage	Outcome Variables	Main Results	Study Risk of Bias
Looney AL et al., 2018 [6]	RCT	Yes	20 dogs	10 to 20 J/cm^2^ (12 Watts, 980 nm, continuous) based on size of dog vs. 0 J/cm^2^ for 6 weeks	Lameness score, Helsinki Chronic Pain Index pain score, NSAID dose	Reduction in NSAID dose, lameness score, pain score in PBMT vs. sham group in dogs with elbow osteoarthritis	Low
Rogatko CP et al., 2016 [7]	RCT	Yes	27 dogs	Single preoperative treatment 3 J/cm^2^ (6 watts, 800–970 nm dual wavelength, continuous and pulsed) vs. sham treatment	Lameness score, response to manipulation, force plate evaluation, radiographic healing	Increased weight bearing of treated dogs on force plate at 8 weeks after TPLO surgery, no other differences	Low
Renwick SM et al., 2018 [8]	RCT	Yes	95 dogs	Three treatments in a 4 day post-operative period, optional fourth treatment 10–14 days post-op, total joules of 252 to 2280 J (up to 15 watt continuous, 20 watt peak power, 660 nm red [100 mW], 800, 905, and 970 nm infrared, with 10 phases of different pulse frequency vs. placebo of red light (660 nm [4 mW]).	Liverpool Osteoarthritis in Dogs, adjusted Canine Orthopedic Index, radiographic healing index of osteotomy, time to cessation of NSAID administration, wound healing	Gait section of adjusted Canine Orthopedic Index improved in laser group compared to control group 8 weeks after TPLO surgery. No other differences.	Low
Kennedy KC, et al. 2018 [9]	RCT	Yes	12 dogs	2.25 J/cm^2^ during hospital treatment and 1.5 J/cm^2^ during at-home treatments (class 2 laser 635 nm) vs. control group treated with the same laser units, with the 5-mW diodes replaced with red LED light-bulbs.	Accelerometers, Canine Brief Pain Inventory, force plate evaluation, radiographs, synovial fluid inflammatory markers	Improved ground reaction forces and pain scores in control group compared to laser group	Low
Santiago V et al. 2014 [10]	Case control experimental	Yes	11 dogs	Group 1: of 90 to 120 J/cm^2^, 790 to 904 nm. Group 2: no laser	Histologic specimens of palatal suture after maxilla expansion	Significant difference in the quality of palatal sutures visually observed. No difference in number of osteoblasts.	Moderate
Wozniak P et al., 1997 [11]	Case Study	No	16 dogs	Details lacking other than continuous 810 nm wavelength	Clinical, radiographic and hematologic clinical assessments	Authors state that most dogs had a positive result of photobiomodulation in dogs with clinical signs of osteomyelitis	High
Lindholm AC et al., 2002 [12]	RCT	Comparison group	179 horses	60 J/cm^2^ (25 watts, 10,600 nm) treatments on days 1, 3 and 5 vs. 12 mg betamethasone and 20 mg HA in affected fetlock joints	Lameness score, flexion test, radiographic and ultrasonographic evaluations	Carbon dioxide laser treatment had 80% response rate vs. 68% for betamethasone/hyaluronic acid treatment for arthritis of the fetlock joint	Moderate
Bergh A, et al. 2006 [13]	RCT	Yes	16 horses	91 J/cm^2^ (16 watts), 5 treatments over 1 week	Lameness score, accerelrometer, synovial fluid inflammatory markers	No significant differences in lameness scores or synovial fluid inflammatory markers	Low
Zielinska P, et al. 2020 [14]	RCT	Yes	25 horses	16 J/cm^2^ (808 nm, 5 watts, 700 Hz) and 16 J/cm^2^ (980 nm, 4 watts, 1000 Hz) vs. control group	Pain, swelling, lameness, ultrasound evaluation	Laser therapy improved pain, edema, lameness, and lesion percentage, but not tendon lesion echogenicity	Low
Marr CM et al., 1993 [15]	Retrospective Study	No	73 horses	Laser compared with polysulfated glycosaminoglyans or conservative therapy	Return to work, time out of training	No significant differences among groups regarding return to work or time out of training	High
Bergh A et al., 2006 [16]	Controlled prospective study	Yes	10 horses	91 J/cm^2^ (10,600 nm, 16 watts, continuous)	Skin and muscle temperature, blood perfusion	Laser treatment increased perfusion and skin temperature in both clipped and unclipped skin, with greater increases in clipped skin. No changes in muscle perfusion or temperature.	Low
Godlewska M et al., 2020 [17]	Prospective study	No	16 horses	20 J/cm^2^ (600 J total, 808 nm, 4 watts, 700 Hz) and 15 J/cm^2^ (980 nm, 450 J total, 2000 Hz)	Surface temperature measurements obtained by thermography camera	Surface temperature of fetlock joints increased by a mean of 3.0 degrees C after laser treatment	Low
Monici M et al., 2018 [18]	Ex-vivo experimental study	No	Cadaveric specimens from 1 horse	Two laser diodes, 905 nm (25 watt peak power with 100 ns pulse width, 10 Hz) and 808 nm (1.1 watt continuous mode)	Laser penetration measured in up to 50 μm thickness sections of tendon and ligament with pyroelectric detector	Penetration depth values for superficial digital flexor tendons and suspensory ligaments were 0.5 mm and 0.16 mm, respectively, corresponding to a respective power attenuation factor of 10^−3^/mm and 10^−4^/mm, respectively	Moderate

**Table 2:** Summary of laser and LED therapy based on a systematic review of the published literature: skin and wound healing.

Skin and Wound Healing							
Study	Study Design	Control Group	Study Sample	Intervention and Dosage	Outcome Variables	Main Results	Study Risk of Bias
Gammel JE et al., 2018 [19]	RCT	Yes	10 dogs	5 J/cm^2^ (980 nm, 2 to 3.5 watts, daily treatment for 5 days) vs. sham treatment	Visual assessment, wound measurements, photographs, and biopsies of surgical incisions and punch biopsy wounds	No differences between groups regarding subjective assessment of healing time and wound measurements. Control group had more necrosis and perivascular lymphocytes and macrophages at day 7, and treated group had more perivascular lymphocytes and macrophages on day 14	Low
Kurach LM et al., 2015 [20]	RCT	Yes	10 dogs	1.125 J/cm^2^ (635 nm, 7.5 mW) 3 times weekly for 32 days vs. standard of care management	Wound planimetry, percent contractions and epithelialization, histologic evaluation	No differences between treated and control wounds for any parameter	Low
Debraekt MM et al., 1991 [21]	RCT	Yes	30 dogs	1 J/cm^2^ (830 nm, 30 mW) 3 times per week for 10 treatments vs. sham treatment	Wound areas and contraction from standard photographs of Von Langenbeck’s palatal repair	No significant differences in quality or rate of wound healing	Low
Wardlaw JL et al., 2019 [22]	RCT	Yes	9 dogs	8 J/cm^2^ (850 nm laser, pulsed 8 Hz with 90% on, 10% off emissions, and 670 nm LED) daily for 7 days vs. non-laser treated controls	Digital photographs and incision healing scores following intervertebral disc surgery	Laser treatment resulted in improved scar scale and cosmetic wound healing	Moderate
Bharti, B et al. 2013 [23]	RCT	Yes	18 dogs	3 J (10 Hz for 5 min) or 6 J (20 Hz for 10 min) daily for 5 days vs. dressing and antiseptic liquid	Biopsies on days 0 and 14	No differences between treated and control groups in histopathological examinations	Moderate
Marchegiani, A. et al., 2019 [24]	RCT	Yes	36 dogs	Blue LED device with peak wavelength between 440 and 460 nm, power density of between 55 and 129 mW/cm 2, for 2 min and antibiotic vs. antibiotic control treatment	Global lesion score, neutrophil engulfing bacterial score for evaluation of canine interdigital pyoderma	Significant improvement in both scoring systems compared to antibiotic control group, and decreased time to resolution of lesions	Moderate
Stich AN et al., 2014 [25]	RCT	Yes	30 dogs	4 J/cm^2^ (980 nm and 810 nm dual wavelength with 80%/20% output, 4 W, 3 times per week for 2 weeks, then 2 times per week for 2 weeks) vs. placebo laser	Localized atopic dermatitis severity score and owner localized pruritic visual analog score in dogs with atopic dermatitis	No significant differences between groups from weeks 0 and 5, but both groups had decreased scores from baseline	Low
Kampa N et al. 2020 [26]	Controlled trial	No	24 dogs	4 J/cm^2^ (830 nm, 200 mW) continuous and pulsed laser with contact and non-contact application	Power meter to determine penetration depth through abdominal skin	Continuous laser had higher mean output power than pulsed laser, mean output power greater for contact vs. non-contact technique, tissue penetration up to 14 mm of tissue depth	Low
Hochman-Elam LN et al., 2020 [27]	Prospective research study	N/A	47 dogs	Two laser systems used, Class IV (980/810 nm, 0.5, 1,5, 3, and 5 W assessed) and Class IIIb (904 nm, 500 mW)	Thermophile laser sensor to detect light penetration through inguinal fold and calcaneal tendon in different coloured dogs with varying coat lengths	Laser transmission was greater in Class IV laser, higher powers, dogs with shaved skin, less skin pigment. Coat length was not a significant predictor of laser penetration.	Low
Keerti N et al., 2016 [28]	RCT	Yes	12 dogs	2.4 J (30 Hz, 2 or 4 min) for 7 days vs. antibiotic control	Visual score cards for evaluation of inflammation, exudation, embedding of sutures, and gross appearance of suture line in dogs with aural hematomas	Laser treated dogs had less inflammation, exudation, minimum embedding of sutures and faster healing	High
Olivieri L et al., 2014 [29]	Case study	No (untreated area of skin for comparison)	7 dogs	3 J/cm^2^ (3 diodes, 470 nm, 685 nm, 830 nm, 13 × 16 MW, 4 × 50 mW, and 4 × 200 mW, respectively, 5 Hz) twice weekly	Hair regrowth in dogs with canine non-inflammatory alopecia	Hair regrowth greatly improved in 6/7 dogs, improved in 1	High
Kaneps AJ et al., 1984 [30]	RCT	Yes	6 horses	57 mJ, (904 nm, pulsed with 300 nsec pulse dura-tion), 15 min, for 5 days	Histologic evaluation of skin and superficial digital flexor tendons of horse with surgical incisions	No qualitative differences in healing between laser irradiated and nonirradiated tissues	Low
Petersen SL et al., 1999 [31]	RCT	Yes	6 horses	2 J/cm^2^ (830 nm, 30 mW) daily vs. nontreated controls	Photoplanimetry to evaluate wound contraction and epithelialization on surgically created open wounds	Laser had no clinically significant effect on wound healing	Low
Gomez-Villamandos RJ et al., 1995 [32]	RCT	Yes	12 horses	He-Ne fibroendoscope, daily for 7 days	Histopathology of created pharyngeal mucosal ulcers	Laser treatment accelerated cicatrisation faster than controls	Moderate
Bader OA et al., 2011 [33]	RCT	Yes	40 wounds	9.72 J/cm^2^ laser treatment compare with bandage alone, copper sulfate ointment, silver nitrate ointment, red mercury ointment	Clinical and histopathology of created wounds with granulation tissue	Surgical removal of granulation tissue followed by laser treatment resulted in more rapid healing	Moderate
Bergh A, 2007 [34]	RCT	No	13 horses	91 J/cm^2^ to skin of hamstrings, 137 J/cm^2^ to fetlock, and 450 J/cm^2^ to loin areas (10,600 nm, 16–20 W)	Histology of skin in horses	Dose dependent changes occurred in skin histology after laser, with severe tissue damage in the 450 J/cm^2^ dose	Low
Duesterdieck-Zellmer KF et al., 2016 [35]	Research Study	No	19 equine cadavers	1 W, 800 and 970 nm	Percentage of energy penetration by photodetector measured in superficial digital and deep digital flexor tendons before and after clipping and after shaving in horses of different skin color	Clipping or shaving improved laser penetration, light-colored skin allowed the greatest penetration, 800 nm was best in light-colored skin, and 970 nm best in dark-colored skin. Only 1–20% and 0.1–4% of energy was absorbed by SDFT and DDFT, respectively	Low
Ryan T et al., 2007 [36]	Research Study	No	9 equine cadavers	500 mW, 810 nm	Photodetector for measuring penetration of laser in tendons	Coat colour did not affect penetration to superficial digital flexor tendons. Clipped hair and skin cleaned with alcohol increased light transmission	Low
Luna SPL et al., 2020 [37]	Experimental study	No	12 horses	2 lasers compared, 67.92 J/cm^2^ (980 nm, 9 W) and 0.34 J/cm^2^ (905 nm, superpulsed peak power 50 W, 1.25 W average output)	Cervical skin thickness measured with a cutometer and ultrasound, percentage of laser penetration measured with photodector	There was greater penetration in cervical skin with the superpulsed laser than the class IV laser. There was also greater penetration in light skin horses	Moderate

**Table 3:** Summary of laser and LED therapy based on a systematic review of the published literature: pain.

Pain							
Study	Study Design	Control Group	Study Sample	Intervention and Dosage	Outcome Variables	Main Results	Study Risk of Bias
Tomacheuski RM et al., 2020 [38]	RCT	No	16 dogs	10 J over 5 acupuncture points (904 nm, 124 Hz) laser acupuncture vs. meloxicam	Glasgow Composite Measure Pain Scale and Dynamic Interactive Visual Analog Scale	Dogs with laser acupuncture had lower pain scores at several time points following routine ovariohysterectomy	Moderate
Nascimento FF et al., 2019 [39]	RCT	Yes	30 cats	3 J/cm^2^ (904 nm, 70 mW, 124 Hz) at acupuncuture points vs. electroacupuncture at same points vs. control	Interactive Visual Analogue Scale and UNESP-Botucatu Multidimensional Composite Pain Scale in cats undergoing routine ovariohysterectomy.	The pain scores did not significantly differ between the treatment groups at any time point. The prevalence of rescue analgesia was significantly higher in the control group than in the laser acupuncture and electroacupuncture groups	Moderate
Martin BB et al., 1987 [40]	Case study	No	14 horses	3 mW, 904 nm, 360 Hz, 2 min contact over each of five acupuncture points once weekly for 8–16 weeks	Clinical signs of back pain, ability to perform, owner evaluation of horses with back pain	Clinical signs alleviated in 10, 3 unchanged, 1 lost to follow-up	High
Haussler KK et al., 2020 [41]	RCT	No	61 horses	94 J/cm^2^ (4 810 nm diode lasers, 3 W total power) laser applied to 5–10 sites based on clinical findings compared with chiropractic or combined laser and chiropractic treatment	Visual analog scale of perceived back pain and dysfunction, detailed spinal examinations evaluating pain, muscle tone, and stiffness.	Laser therapy produced significant reductions in back pain, epaxial muscle hypertonicity, and trunk stiffness. Combined laser and chiropractic produced similar reductions. Chiropractic treatment by itself did not produce any significant changes in back pain, muscle hypertonicity, or trunk stiffness.	Moderate
Brevault S et al., 2016 [42]	RCT	Yes	Details unavailable	Details unavailable	Muscle tone, back mobility, deep and superficial sensitivity, dynamic assessment and rider evaluation of back pain in horses	Muscle tone, back mobility, deep and superficial sensitivity improved with laser treatment, but no differences in dynamic assessment and rider evaluation	High
Petermann U, 2011 [43]	Case study	No	21 horses	Details unavailable, laser therapy to acupuncture points	Pain score of horses with laminitis	Post treatment pain scores improved over pre-treatment scores	High
Ghazaleh N et al., 2018 [44]	Prospective experimental cross-over design	Yes	5 horses	Laser treatment (3000 Hz for 10 min) compared with saline, lidocaine, and laser plus lidocaine caudal epidural injections	Motor and sensory blockade evaluations assess by transcutaneous electrical nerve stimulation, noxious stimulus with a pin, and pinch test in horses undergoing epidural analgesia	No difference in sensory or motor stimulation response between groups, but laser in combination with lidocaine had a longer duration of analgesia than laser or epidural alone	High

**Table 4:** Summary of laser and LED therapy based on a systematic review of the published literature: neurologic conditions.

Neurologic Conditions							
Study	Study Design	Control Group	Study Sample	Intervention and Dosage	Outcome Variables	Main Results	Study Risk of Bias
Bruno E et al., 2020 [45]	Retrospective study	Yes	24 dogs	4 J/cm^2^ (808 and 905 nm, 50% duty cycle, 18 Hz,1.2 W with peak power of 75 W, correction of dose with skin color) daily at the time of rehabilitation vs. rehabilitation and no laser.	Modified Frankel scoring system in dogs undergoing postoperative care following intervertebral disk herniation surgery	There was no statistical difference in time to regain ambulatory ability	Moderate
Bennaim M et al., 2017 [46]	RCT	Yes	32 dogs	12 J (810 nm, 1 W cluster probe with 5 clusters, 5.5 W/cm2, 2.5 Hz) daily for 5 days vs. laser and physical rehabilitation with sham laser or sham laser only	Duration of postoperative IV opioid administration and recovery grades in dogs following intervertebral disk herniation surgery	Time to reach recovery and duration of postoperative IV opioid administration did not differ among groups	Low
Williams CC et al., 2011 [47]	RCT	Yes	17 dogs	635 nm, 9–1151 Hz, other details not available vs. control	Recovery time following intervertebral disk herniation surgery	Laser did not shorten recovery times	Moderate
Draper WE, et al. 2012 [48]	RCT	Yes	36 dogs	12 J (810 nm, 1 W cluster probe with 5 clusters, 25 W/cm^2^) over 3 sites for 1 min/site daily for 5 days vs. no treatment	Modified Frankel scoring system in dogs following intervertebral disk herniation surgery	Time to achieve a modified Frankel score of 4 was significantly lower in the laser group compared to the control group	Moderate
Sharifi, D et al. 2005 [49]	Experimental controlled study	Yes	10 dogs	Details not available regarding laser treatment except 10 min of laser treatment daily for 2 weeks vs. no laser	Electromyography (EMG)	EMG showed significant differences in muscle force of the semimembranosus and semitendinosus muscles in dogs undergoing experimental crush sciatic nerve injury	High
Miller LA et al., 2020 [50]	Retrospective	No	20 dogs	Class III laser 8 J/cm^2^ (904 nm, 500 mW) vs. Class IV laser 14–21 J/cm^2^ (980 nm, 6–12 W)	Time from symptom onset and euthanasia, time between symptom onset and nonambulatory paresis or paralysis	Dogs receiving Class IV laser had slower disease progression and longer survival times	High

## Data Availability

Not applicable.
